# The effect of attention bias modification on depressive symptoms in a comorbid sample: a randomized controlled trial

**DOI:** 10.1017/S0033291722003956

**Published:** 2023-10

**Authors:** Ragnhild Bø, Brage Kraft, Mads Lund Pedersen, Jutta Joormann, Rune Jonassen, Kåre Osnes, Catherine J. Harmer, Nils Inge Landrø

**Affiliations:** 1 Clinical Neuroscience Research Group, Department of Psychology, University of Oslo, Oslo, Norway; 2 Division of Psychiatry, Diakonhjemmet Hospital, Oslo, Norway; 3 Department of Psychology, University of Oslo, Oslo, Norway; 4 NORMENT, Division of Mental Health and Addiction, Oslo University Hospital & Institute of Clinical Medicine, University of Oslo, Oslo, Norway; 5 Affect Regulation and Cognition Lab, Yale University, USA; 6 Faculty of Health Sciences, Oslo Metropolitan University, Norway; 7 Department of Psychiatry, Oxford University, UK; 8 Oxford Health NHS Foundation Trust, Warneford Hospital, Oxford, OX3 7JX

**Keywords:** Attention bias modification, depression, follow-up, transdiagnostic

## Abstract

**Background:**

Studies investigating the long-term effect of attention bias modification (ABM) in clinical samples are lacking. This study investigates the 6-months follow-up effect of ABM on depressive symptoms in participant with major depressive disorder with and without comorbid disorders.

**Methods:**

We conducted a double-blind randomized sham-controlled trial in 101 participants between 19 November 2019, and 17 August 2021. Follow-up ended 3 April 2022. Participants were allocated to ABM or sham condition twice daily for 14 consecutive days. Primary outcomes were the total score on the Beck Depression Inventory-II (BDI-II) at 6 months, mean Brief State Rumination Inventory (BSRI) score post-treatment and reduction in BSRI post-treatment. Secondary outcome was change in attentional bias (AB). The trial was preregistered in ClinicalTrials.gov (#NCT 04137367).

**Results:**

A total of 118 patients aged 18–65 years were assessed for eligibility, and 101 were randomized and subjected to intention-to-treat analyses. At 6 months, ABM had no effect on depression and anxiety compared to a sham condition. While rumination decreased during the intervention, there was no effect of condition on rumination and AB. Predictor analysis did not reveal differences between participants with ongoing major depressive episode or comorbid anxiety.

**Conclusion:**

Compared to sham training, there was no effect of ABM on depressive symptoms at 6-months follow-up. Since the intervention failed at modifying AB, it is unclear whether changes in AB are related to long-term outcomes.

## Introduction

Attention bias modification (ABM) has shown some promising results as a low-cost intervention for reducing depression symptoms in clinical groups (Hallion & Ruscio, 2011), but only a small number of RCTs have been conducted and overall effect size estimates are small (Fodor et al., 2020). ABM is a computerized intervention aiming to reduce negative attentional bias (AB). Furthermore, a negative AB is found to be causally related to depressive (Wells & Beevers, 2010), and anxiety symptoms (Van Bockstaele et al., 2014). Depression is often comorbid with other mental disorders (Kessler et al., 1997), and comorbidity is associated with poorer prognosis (Coplan, Aaronson, Panthangi, & Kim, 2015) and more severe psychopathology (Kessler et al., 2005). Treatments targeting transdiagnostic processes, such as negative AB, are therefore needed. ABM paradigms which demonstrate a modification of AB has been found to influence emotional vulnerability (Grafton et al., 2017). ABM may therefore be a viable option for modifying transdiagnostic processes in comorbid samples and lead to symptom reduction

Depression is a disorder characterized by high relapse rates, especially among patients with comorbid disorders (Burcusa & Iacono, 2007), hence treatments should ideally have lasting effects beyond the effect found when terminating treatment. ABM may produce long-lasting effects. For example, a study by Yang, Ding, Dai, Peng, and Zhang (2015) found better outcome in the ABM condition as compared to the waitlist and placebo conditions at 3 months in a sample of students with subclinical depressive symptoms. However, the effect was not evident at 7 months follow-up. The same group (Yang, Zhang, Ding, & Xiao, 2016) also found greater reductions in depressive symptoms for ABM compared to placebo in a sample of adolescents with major depressive disorder (MDD) at 12 months follow-up. de Voogd et al. (2016) found reductions in depressive symptoms among unselected adolescents up to 12 months following ABM or attentional training, but no difference when compared to placebo. Browning, Holmes, Charles, Cowen, and Harmer (2012) reported beneficial effect on depressive symptoms 1 month after ABM in adults with remitted MDD, as did Dai, Hu, and Feng (2019) in their study including patients with clinical depression. However, findings are not equivocally positive. In a large trial combining ABM and interpretation bias, incidence of depression and symptom severity, was similar across the active and the control condition over 12 months (Basanovic et al., 2020). None of the studies reported on comorbidity. Studies examining the effects of ABM tend to have a short follow-up period, often only assessing the effect immediately after the completion of the intervention (see online Supplemental eTable 2 in review by Fodor et al., 2020). This limits our knowledge regarding the long-term effect of ABM for depression.

Many studies are also limited by a low number of ABM sessions, spanning 4–10 sessions (Fodor et al., 2020). According to Blackwell (2020), the low number of sessions contradicts knowledge we have about associative learning, which normally requires more and frequent repetitions to produce lasting effects. A notable exception is a recent study by Basanovic et al. (2020), involving 44 sessions over 52 weeks investigating various depression-related outcomes. In our research group we have examined an ABM paradigm consisting of 28 sessions over 14 consecutive days. This resulted in a small reduction in clinician rated depression (but not in self-reports), immediately after the intervention (Jonassen et al., 2019). The same paradigm was previously found by Browning et al. (2012) to successfully modify AB. Thus, the intensity and frequency of this paradigm seems to be sufficient to induce meaningful change in AB and symptoms.

Building on our former study, the present study includes participants with a broader spectrum of depressive symptoms and comorbid disorders. By targeting a presumed underlying transdiagnostic mechanism, that is negative AB (Garland & Howard, 2013), we investigate whether the effect of this intervention also upholds in a heterogeneous clinical group that reports having problems with depressive symptoms, independent of currently experiencing an ongoing episode of MDD, having comorbid disorders, or not. Negative AB is a trait marker apparent in all phases of MDD (Joormann & Gotlib, 2007), and modification of this cognitive vulnerability marker could both reduce symptom severity and prevent relapse (Browning et al., 2012). Rumination is associated with AB (Hur, Gaul, & Berenbaum, 2019) and is a transdiagnostic mechanism mediating the relation between depression and anxiety (McLaughlin & Nolen-Hoeksema, 2011), and we investigate whether the effect of ABM is mediated by state rumination and change in state rumination. With the long-term aim of introducing ABM as a viable low-threshold treatment option, there is an apparent need for a translational study including long-term follow-up and a representative sample of participants who self-report depressive symptoms. The aim of the current study is to investigate the long-term effect of ABM on depressive symptoms, and whether it is mediated by state rumination, in a sample with and without comorbid disorders in a preregistered trial. Given that symptom reduction is reliant on modification of AB, we predict that ABM will lead to lower levels of depressive symptoms compared to a sham condition at 6-months follow-up.

## Methods

### Study design

The present study is a double-blind randomized controlled trial comparing the effect of ABM *v.* a sham condition between November 2019 and April 2022. The study was preregistered at the US National Institutes of Health (ClinicalTrials.gov) # NCT04137367 and approved by the Regional Committee for Medical and Health Research Ethics (REK SørØst 2019/33).

### Study population

Participants aged 18–65 years with ‘bothersome depression symptoms’ were recruited using local advertisements and social media. Entrance to the trial was via self-referral by phone or online registration. Potential participants were pre-screened by telephone before in-person formal clinical evaluation and enrollment to the trial. Participants gave written informed consent before further evaluation. Demographic information along with information about current and past treatments for mental health, including the current use of psychotropic medication, was obtained. Clinical psychologists or trained psychology students conducted a diagnostic interview using the Norwegian version of the MINI International Neuropsychiatric Interview PLUS 5.0.0 (M.I.N.I.). Inclusion criteria were current or previous MDD, with or without comorbid mental disorders. Exclusion criteria were current or former manic episodes, psychotic episodes, or neurological disorders. It is commonly found that patients experiencing a major depressive episode fulfill the criteria for bipolar disorder (Angst et al., 2011). Therefore, we allowed patients with bipolar II disorder to enter the trial. Since bipolar disorder involves episodes with mood congruent psychotic symptoms, we excluded bipolar I disorder as these symptoms require treatments which targets other mechanisms compared to what ABM may offer.

**Fig. 1.**
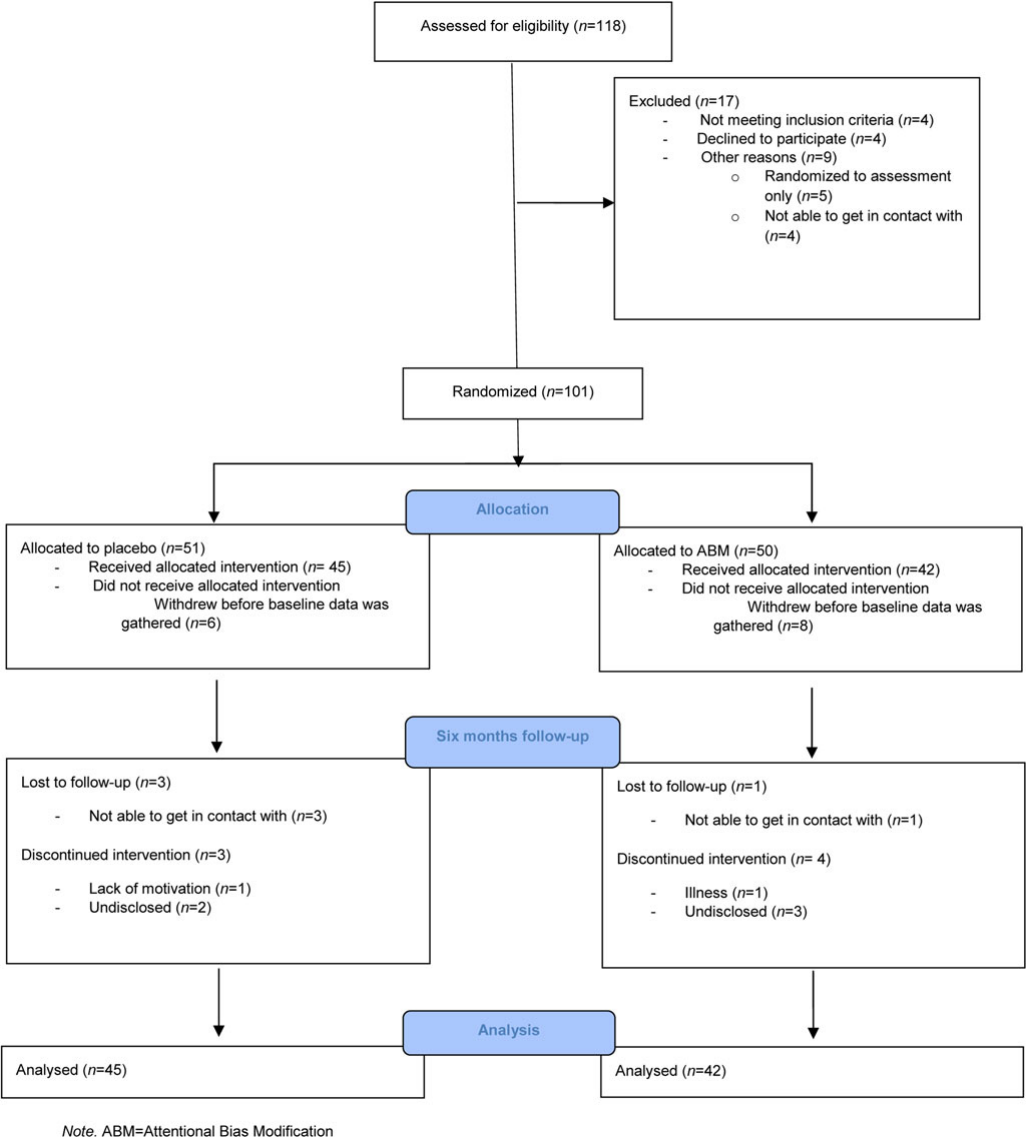
CONSORT flow diagram.

Eligible participants were invited to return for face-to-face baseline assessment 2 weeks later wherein self-reported anxiety symptomatology [Beck's Anxiety Inventory (BAI); Beck, Epstein, Brown, & Steer, 1988a) and self-reported alcohol consumption habits (AUDIT; Saunders, Aasland, Babor, De la Fuente, & Grant, 1993) were assessed. See Fig. 1 for details regarding enrollment.

### Randomization and masking

Allocation to condition occurred once the participant was included in the trial. An independent lab-technician not related to the current investigation conducted a simple randomization of participants by means of lottery draw from slips in a container (1:1 ratio). Both study investigators and participants were blinded to treatment allocation. The code for revealing condition allocation was unlocked in April 2022, after the 6-months follow-up data collection came to an end and after statistical analyses were conducted.

### Study intervention

Participants were randomized to receive either ABM or a sham condition. ABM was a computerized visual dot-probe procedure adopted from Browning et al. (2012) and identical to the one reported in Jonassen et al. (2019). The total number of prescribed sessions was 28 for 14 consecutive days (i.e. twice daily). Each session lasted for approximately 5–7 min and was conducted at home on laptops provided by the research group for this purpose only.

Stimuli consisted of pairs of images of faces displaying emotional expressions: positive (happy), negative (fear or anger) and neutral. Each stimuli pair was derived from two out of three valences (positive-negative, negative-neutral, positive-neutral) displayed horizontally in random order, for either 500 or 1000 ms. Immediately after, a probe (one or two dots) appeared on the computer screen in one of the two locations of the previously displayed stimuli. Participants were instructed to as fast and accurately as possible to indicate the correct number of dots in the probe. Each session consisted of 96 trials with equal number of the three stimuli pairs.

In the ABM condition, the probe appeared in the location of the screen where the relatively more positive stimuli had been displayed for 87% of the trials, and in the location of the relatively more negative stimuli for 13% of the trials. By being exposed to this condition, participants should implicitly learn to deploy their attention to the location of the relatively more positive stimuli, thereby developing a less negative AB. In the sham condition, there was no contingency between probe location and stimuli (i.e. the probe appeared in the location of the screen where the relatively more positive stimuli had been displayed for 50% of the trials). In all other respects, the sham condition was equal to the active condition.

Participants were informed that the trial would investigate the relation between attentional focus and depression. They were also informed about the potential therapeutic properties of ABM and about the randomization procedure but were kept blind of their allocation. However, they were not given any details on the difference between conditions.

According to the preregistration of the trial, the plan was to include an assessment only condition in addition to ABM and the sham condition. However, due to restraints on conducting research during the COVID-19 pandemic, allocation to this condition was discontinued after including five participants. This change to the protocol was registered in clinicaltrials.gov.

### Study outcomes

The primary outcome was the Beck's Depression Inventory-II (BDI-II; Beck, Steer, & Carbin, 1988b) at six-months follow-up. BDI-II is a self-report measure consisting of 21 items assessing depressive symptoms and attitudes on a four-point Likert scale, ranging from 0 to 3. The questionnaire is among the most commonly used scales for assessing depression in clinical research. Cronbach's *α* was: *α* = 0.870 at baseline, *α* = 0.970 post intervention, and *α* = 0.907 at six-months follow-up.

Other primary outcomes were state rumination as measured by the Brief State Rumination Inventory (BSRI; Marchetti, Mor, Chiorri, & Koster, 2018) post-treatment and reduction in BSRI from baseline to post-treatment. As stated in the protocol, the original plan was to investigate whether (change in) state rumination mediated the relation between ABM and BDI-II at 6-months follow-up. The BSRI consists of eight items (e.g. ‘Right now, I am thinking: Why do I have problems other people don't have?’) and is a valid and reliable measure of state rumination. Each item is rated on a 100 mm visual analogue scale ranging from ‘completely disagree’ to ‘completely agree’. Before assessing state rumination, the participants went through a stress induction procedure consisting of listening to a recording of an imagery script that was personalized by autobiographical content (see Bø et al., 2022 for details), based on the procedure described by Sinha and Tuit (2012).

The secondary outcome was decrease in negative AB from baseline to post-treatment as measured by a dot probe task. This task is the same as one session of sham condition. AB was calculated as the difference in reaction time in milliseconds between trials where the probe replaced the relatively more negative face *v.* the more positive face (see Jonassen et al. (2019) for details). Hence, a positive score reflects a relative AB away from negative stimuli, while a negative score reflects a relative AB toward negative stimuli.

### Sample size and statistical analysis

We estimated *a priori* that a total of 100 participants would be needed to detect a difference between ABM and sham condition, with a two-tailed *α* of 0.05 and (1-*β*) of 0.80. Power calculation was based on the assumption that 50% of the participants allocated to ABM would report a minimum of 3 points reduction on BDI-II at 6 months, compared to 20% of the participants allocated to the sham condition. In comparison, in the Jonassen et al. (2019) study, 45.1% of the participants in the active condition reported reductions in depression symptom of this magnitude. That sample consisted of participants with MDD in remission and comparatively lower symptom levels, thus justifying a slight increase in the proportion of responders in this trial due to larger opportunity for improvement. According to the National Institute of Health and Clinical Excellence (2009), a difference in ⩾3 BDI-II points is considered a clinically meaningful effect of depression treatment. Assuming that 15% of participants would be lost to follow-up, power calculation indicated the need for 50 participants in each condition.

Baseline characteristics of participants in the two treatment groups were reported using frequency distributions and descriptive statistics including measures of central tendency and dispersion. The primary analysis was conducted using an intention-to-treat approach and included all randomized participants who had either baseline and/or follow-up data (*n* = 87). Primary outcomes (BDI-II at six-months follow-up, BSRI post training, and change in BSRI from baseline to post-intervention), secondary outcome (change in AB), were analyzed using four mixed-model analyses with random intercepts. For exploratory purposes we investigated the effect of ABM on anxiety (BAI). We also conducted a predictor analysis, including the interaction effect between Ongoing MDD/Comorbid anxiety × Assessment point × Condition in the analysis of BDI-II. This is presented in the online Supplementary materials.

For BDI-II and BAI, the model included condition (ABM, sham training) and assessment point (baseline, post ABM, six-months follow-up) and condition by time interaction as fixed factors. Due to the different intervals between assessment points, we treat time as a categorical variable. The same specifications were used for the analysis of BSRI and AB but included two time points only (baseline and post ABM). The model was fit using restricted maximum likelihood using the lme4 package in R (Bates, Mächler, Bolker, & Walker, 2015). The ABM treatment effect was operationalized as the least squares mean difference at six-months from this mixed-model repeated measure model. Statistical significance was assessed using the LS mean *p* value < 0.05.

If a Condition × Assessment point interaction effect [or in the case of predictor analyses (ongoing MDD or comorbid anxiety disorder): Predictor × Condition × Assessment point] was significant, pairwise analysis was used for decomposing the interaction effect. In case of an effect of ABM, we investigated whether state rumination and change in state rumination mediated the effect.

At baseline, 15 participants had missing data on BDI-II, AB, and BAI and 14 participants had missing BSRI data. Participants with missing data at baseline, except for one who had follow-up data, were not included in the analysis. Missing data on follow-ups had the following distribution at various timepoints; BDI-II post-intervention: ABM *n* = 13, sham condition *n* = 10; BDI 6-months follow-up: ABM: *n* = 13, sham condition *n* = 12; BSRI post-intervention: ABM *n* = 12, sham condition *n* = 9; AB post-intervention: ABM *n* = 15, sham condition *n* = 9; BAI post-intervention: ABM *n* = 13, sham condition *n* = 10; BAI 6-months follow-up: ABM *n* = 13, sham condition *n* = 12. Missing data were not imputed as analysis with the mixed-model approach without any ad hoc imputation is shown to be more powerful compared to other alternatives (Chakraborty & Gu, 2009).

All analyses were conducted using SPSS (version 27) and R (version 4.2.0).

### Role of the funding source

The funders of the study had no role in study design, data collection, data analysis, data interpretation, or writing of the report.

## Results

### Study population

A total of 118 adults were screened for eligibility, and 101 met eligibility criteria and were randomized between November 2019 and September 2021 (Fig. 1). See Table 1 for baseline demographic and clinical characteristics between participants who were allocated to ABM or a sham condition. Forty-seven participants fulfilled criteria for MDD, 96 for previous MDD, 24 for dysthymia, two for hypomania, 12 for previous hypomania, 14 for panic disorder, 18 for agoraphobia, 31 for social phobia, seven for obsessive-compulsive disorder, eight for post-traumatic stress disorder, 20 for alcohol use disorder, seven for substance use disorder, and 40 for generalized anxiety disorder. The median number of current mental disorder was three. Seventy-six participants completed the trial and provided data at six-months follow-up.

**Table 1.** Baseline characteristics of all randomized participants

	Placebo (*n* = 51)	ABM (*n* = 50)
Sex		
Female	41 (80%)	31 (62%)
Male	10 (20%)	19 (38%)
Age (years)^a^	44 (11.3)	44 (10.3)
Education level (ISCED)^a^	5.7 (1.3)	5.6 (1.3)
Ongoing MDD	20 (39%)	27 (54%)
Previous MDD	48 (94%)	48 (96%)
Current SSRI	11 (22%)	16 (32%)
Current comorbid anxiety disorder	37 (73%)	34 (68%)
Baseline characteristics		
BDI-II^a^	26.3 (9.8)	23.1 (10.2)
BAI^a^	16.2 (8.9)	13.1 (8.8)
AUDIT^a^	5.5 (5.6)	5.8 (4.7)
BSRI^a^	47.8 (20.6)	45.7 (21.4)
Attentional bias (*ms*)^a^	−5.1 (33.8)	−10.2 (23.9)
Number of sessions completed	19.9 (6.8)	20.0 (7.2)
Number of days from intervention to 6-months follow-up	199.7 (28.2)	194.6 (14.7)

ABM, attentional bias modification; AUDIT, Alcohol Use Disorder Identification Test; BAI, Beck's Anxiety Inventory; BDI-II, Beck's Depression Inventory-II; BSRI, Brief State Rumination Inventory; ISCED, International Standard Classification of Education; MDD, major depressive disorder; SSRI, participants currently using an antidepressant belonging to the selective serotonin reuptake inhibitors.

*Note.* Data are *n* (%) or *mean* (s.d.).

a Data not available for all randomized participants. See text for details.

While potential baseline differences between study arms were not subjected to significance testing, as the differences per definition should be at random due to randomization procedure, we note that the difference in self-reported depression scores correspond to what has been deemed as clinically meaningful (3.2 BDI-II points; NICE, 2009). Prior depression scores are highly predictive of future depression scores, and the random intercepts in the mixed-model analysis takes into account this difference when assessing outcomes.

### Primary outcome

#### Depressive symptoms

**Fig. 2.**
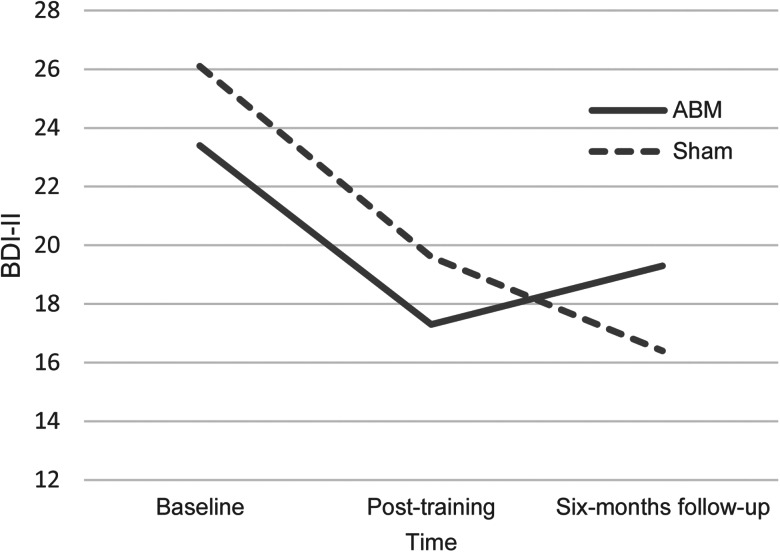
Estimated self-reported symptom levels for ABM and placebo at baseline, post-training, and six-months follow-up. *Note*. *N* = 87. BDI-II, Becks Depression Inventory-II.

Compared to baseline, there was a significant reduction in depression from baseline to post-treatment, *t* (196.466) = −12.85, *p* < 0.001, *d* = −1.84, from baseline to six-months follow-up, *t* (202.100) = −10.63, *p* < 0.001, *d* = −1.50, and from post-treatment to six-months follow-up *t* (163.4632) = −10.09, *p* < 0.001, *d* = −1.58. There was no main effect of ABM condition, *t* (172.375) = 1.34, *p* = 0.18, *d* = 0.20. There was no significant interaction effect between ABM and assessment point from baseline to post-treatment, *t* (168.810) = 0.018, *p* = 0.86, *d* = 0.03, but there was a significant interaction effect between ABM and assessment point from baseline to six-months follow-up, *t* (169.170) = 2.54, *p* = 0.012, *d* = 0.39, and from post-treatment to six-months follow-up, *t* (150.8753) = 2.25, *p* = .026, *d* = 0.37. The interaction effect was in the opposite direction to what was predicted, that is reduction in the sham condition, but not in the ABM condition. The results showed a similar reduction in depression from baseline to post-treatment for both groups, and that the symptom development differed between ABM and sham condition at longer follow-ups, and after treatment termination. At six months, the difference between groups was not significant, *F*_(1,74)_ = 0.876, *p* = 0.38, *η^2^* = 0.20 (see Fig. 2). Due to the lack of effect, the planned mediation analysis was not performed.

Conducting pairwise comparisons of assessment points within groups, we found that in the sham condition, there was a significant reduction from baseline to post-training, *t* (40) = 4.32, *p* < 0.001, *d* = 0.68, and from baseline to six months, *t* (38) = 5.21, *p* < 0.001, *d* = 0.83, but no significant change from post-training to six-months follow-up, *t* (37) = 1.57, *p* = 0.126, *d* = 0.25. In the ABM group there was a significant reduction from baseline to post-training, *t* (35) = 5.78, *p* < 0.001, *d* = 0.91, and from baseline to six months, *t* (35) = 2.280, *p* = .029, *d* = 0.38, but no significant change from post-training to six-months follow-up, *t* (35) = −1.250, *p* = 0.220, *d* = −0.21.

See online Supplementary material for predictor analysis for the effect of ongoing MDD and comorbid anxiety, respectively.

#### State rumination

In BSRI scores, there was a main effect of assessment point, suggesting decrease in state rumination from pre- to post-training, *t* (80.69) = −2.41, *p* = 0.02, *d* = −0.54. There was no main effect of condition, *t* (124.34) = 0.50, *p* = 0.62, *d* = 0.09, nor a significant Assessment point × Condition interaction effect, *t* (81.11) = −1.20, *p* = 0.23, *d* = −0.27. Estimated mean in the active condition was 32.3 and 38.3 in the sham condition. At post-intervention, mean BSRI score in the active group was 34.6 and 32.1 in the sham group. The difference was not significantly different, *t* (70.692) = −0.47, *p* = 0.64, *d* = −0.11.

### Secondary outcome

In AB, there was no main effect of assessment point, *t* (78.41) = 1.52, *p* = 0.13, *d* = 0.34, hence AB was not significantly changed from pre- to post-treatment. There was no main effect of condition, *t* (158.70) = −1.07, *p* = 0.28, *d* = −0.17. There was no significant Assessment point × Condition interaction effect, *t* (80.38) = 1.08, *p* = 0.28, *d* = 0.24. Estimated mean AB score in the active condition was −10.73 at baseline and 6.06 post-intervention, and for the sham condition −4.75 at baseline and 3.46 post-intervention. The difference was not significantly different, *t* (75) = −0.68, *p* = 0.5, *d* = −0.16.

### Exploratory outcome

For exploratory purposes, we investigated if there was an effect of ABM on anxiety, as measured by BAI. Compared to baseline, there was a significant reduction in anxiety from baseline to follow-up *t* (145.24) = −3.51, *p* < 0.001, *d* = −1.80, and from baseline to 6-months follow-up *t* (145.92) = −5.35, *p* < 0.001, *d* = −0.89, but not from post-treatment to six-months follow-up *t* (143.612) = −1.88, *p* = 0.06, *d* = −0.38. There was no main effect of ABM, *t* (125.45) = 1.72, *p* = 0.09, *d* = 0.31. There was no significant interaction effect between ABM and assessment point from baseline to post-treatment, *t* (146.257) = −0.74, *p* = 0.46, *d* = −0.12, but there was a significant interaction effect between ABM and assessment point from baseline to six-months follow-up, *t* (143.240) = 2.25, *p* = 0.03, *d* = 0.38, and from post-treatment to 6-months follow-up, *t* (146.57) = 3.01, *p* < 0.01, *d* = 0.50. The results indicate a similar reduction in anxiety from baseline to post-treatment for both groups, and that the symptom development differs between groups at longer follow-ups and after treatment termination, where the sham condition continues reductions, while the ABM condition does not.

Pairwise comparisons of assessment points within groups, showed that in the sham condition, there was a significant reduction from baseline to post-training, *t* (40) = −4.87, *p* < 0.001, *d* = −0.76, and from baseline to 6 months, *t* (38) = −4.36, *p* < 0.001, *d* = −0.70, but no significant change from post-training to six-months follow-up, *t* (37) = 1.34, *p* = 0.17, *d* = 0.23. In the ABM condition, there was a significant reduction from baseline to post-training, *t* (35) = −3.79, *p* < 0.001, *d* = 0.63, but not from baseline to six months, *t* (35) = 0.83, *p* = 0.41, *d* = 0.14, and from post-training to six-months follow-up, *t* (35) = −1.19, *p* = 0.24, *d* = −0.20. The difference between conditions at 6 months was not statistically significant, *F*_(1,74)_ = 1.39, *p* = 0.41, *η^2^* = 0.19 (see online Supplementary Table S3).

For exploratory purposes, we investigated if change in AB (post-intervention AB–baseline AB) was associated with change in depressive symptoms from baseline to post-intervention (post-intervention BDI-II–baseline BDI-II) and six-months follow-up (six-months follow-up BDI-II–baseline BDI-II), respectively. Correlation analyses showed no association between change in AB and change in BDI-II symptoms between either timepoints in either group, or in groups combined, all *r*'s < 0. 17, *p*'s > 0.3.

## Discussion

### Main findings

The current study examined the long-term effect of ABM *v.* a sham condition for depressive symptoms in a sample with and without comorbid disorder. At six-months follow-up, there were no significant differences for either depression or anxiety symptoms. Both groups reduced depression levels during the 14 days training period, but no further reduction was found during six-months follow-up. State rumination was decreased during intervention, but there were no significant differences between conditions. Attention bias was not significantly reduced. Predictor analysis of the effect of ongoing MDD and comorbid anxiety, respectively, on depression symptoms, did not reveal any difference.

While we did find a general reduction in self-reported depression, anxiety symptoms, and state rumination over the course of this trial, the add-on benefit of this intervention compared to the natural course of symptoms is unknown due to lack of data from the discontinued assessment only group.

The intervention failed to modify AB which is the proposed mechanism of ABM. It has been argued that modification of AB is a requirement for ABM to have the intended effect on symptoms (Grafton et al., 2017), hence an effect on symptoms in the ABM group might not be expected. Therefore, the current trial was not able to elucidate the relation between reduced AB and long-term depression severity. In general, long-term follow-ups within depression research are challenging, considering that the depressive episodes for the majority (63%) of the general MDD population ultimately tend to reside within six months (Spijker et al., 2002), and the reliance on mean values might have masked different trajectories in depression symptoms.

There is currently no available evidence to support the effectiveness of ABM in long-term reduction of depressive symptoms in adult populations (see also Basanovic et al., 2020; Ferrari, Becker, Smit, Rinck, & Spijker, 2016 for an ongoing trial). However, the current trial failed to have an effect on AB. Therefore, it might be premature to abandon ABM for long-term reduction in depressive symptom based on this study, in particular since the long-term effect of ABM for depressive symptoms in adolescence shows equivocal effects (Yang et al., 2015, 2016). In summary, the current trial brings the overall effect sizes of ABM for depressive symptoms closer to zero.

After ABM, the development of depressive symptoms up to six months was significantly different among groups. While the change within groups was insignificant, the depressive symptoms among the active condition increased, while it continued to decrease among the sham condition. We speculate that the different trajectories might be related to a withdrawal effect from ABM prior to complete recovery. Pertaining to research on antidepressants, there are some indications for a rebound effect, i.e. higher relapse rates or especially severe relapses of depression after the discontinuation of an antidepressant (Henssler, Heinz, Brandt, & Bschor, 2019), and we cannot exclude the possibility of a rebound effect among the participants in the ABM group after treatment termination. If this be the case, a tapering period might be required.

This study took place during the covid-19 pandemic and lockdowns, and we do not know how this affected the outcome of this trial. Implicit tuning of AB through ABM may require face-to-face contact to reach its full potential (Godlewska & Harmer, 2021). Since direct social contact was restricted during the pandemic, it might, although speculative, have limited the effect of ABM.

So why did the intervention fail at modifying AB? The intervention has previously been successful in modifying AB (Browning et al., 2012). The ABM intervention included fearful and angry facial expression only, and sad facial expressions might perhaps have had greater relevance to depression. On the other hand, there has been extensive criticism pointed at the reliability of the dot probe task in measuring AB (e.g. Meissel et al., 2022), and we cannot exclude the possibility that this affects the sensitivity of this measure in this study also.

### Limitations

Measures of depressive symptoms should ideally also include a clinician-rated scale (Uher et al., 2012). Given that a previous study only found a reduction in clinician-rated (and not self-reported) depressive symptoms (Jonassen et al., 2019), including a clinician-rated scale could unveil an ABM effect. Several participants withdrew shortly after randomization; hence, baseline BDI-II was missing for several of the participants. This is a limitation and suggests the need for setting up data collection as to ascertain the recording of important data points at the earliest occasion.

Despite the randomization procedure there was a difference in depressive symptoms at baseline which was clinically meaningful (3.2 BDI-II points). Moreover, current MDD was more prevalent in the ABM group. The therapeutic effect of ABM might be dependent on depression severity and the presence of a diagnosable disorder (Baert, De Raedt, Schacht, & Koster, 2010), though symptom severity was not found to moderate the effect of ABM among patients with remitted MDD (Bø et al., 2021). The predictor analyses, investigating the interaction effect between intervention and ongoing MDD/comorbid anxiety disorders, did not reveal any differences based on depression status nor comorbidity. However, analyses were not *a priori* specified and not subjected to power calculations, nor was randomization procedures stratified for this purpose. The current trial most likely lacks power to detect any but very large predictor effects.

The lack of an assessment-only condition limits interferences of the added benefit of taking part in a structured daily cognitive activity beyond the natural course of depressive symptoms. Moreover, the closely matched control condition of the present trial might be better apt at delineating mechanism in basic research than investigating the effectiveness of ABM in clinical setting (Blackwell, Woud, & MacLeod, 2017), and future translational studies should reconsider the use of sham training as control condition.

## Conclusion

This trial showed that there was no effect of ABM on depressive symptoms at six-months follow-up. These results bring the overall effect sizes of ABM as a depression intervention further towards zero and question the presence of long-term effects. However, since the intervention failed to impact the presumed mechanism of the intervention (AB), we still do not know whether successful modification of AB is related to depressive symptoms in the long-term.

## Supplementary material

For supplementary material accompanying this paper visit https://doi.org/10.1017/S0033291722003956. click here to view supplementary material
