# Supplementary material for: The effect of attention bias modification on depressive symptoms in a comorbid sample: a randomized controlled trial
Source: Psychol Med. 2023 Jan 9;53(13):6389–96. doi: 10.1017/S0033291722003956 (PMC10520597; doi:10.1017/S0033291722003956)
Supplement: Supplementary file 1 [file S0033291722003956sup001.docx]

Supplementary materials

### Ongoing Major depressive disorder (MDD)

We expanded the analysis of the primary outcome by adding ongoing MDD as predictor of treatment effect. Results showed that the main effects and interaction effects of the former analysis remained unchanged. There was a main effect of ongoing MDD, t (154.84) = 2.04, *p* = .04, *d* = .33, but not an interaction effect of ABM x ongoing MDD, t (156.13) = .064, *p* = .53, *d* = .01. There was no significant three-way interaction effect between ABM, ongoing MDD and assessment point from baseline to post-treatment t (151.66) = -1.40, *p* = .17, *d* = .23 nor from baseline to six months follow up t (152.18) = -1.20, *p* = .23, *d* = .19, or from post-treatment to six months follow-up t (148.667) = .018, *p* =.9, *d* = .002. There was no significant difference between groups at six-months follow-up, F (3,75) = 2.1, *p* = .11, *η^2^*= .08. Results suggests that whether participants are in remission or have ongoing MDD, does not moderate the relation between ABM and depressive symptoms at any of the assessment points.

***Supplementary table 1****.*

Estimated marginal means of depression severity as measured by BDI-II separated by ongoing MDD.

|  | Baseline | Post-intervention | Six-months follow-up |
| --- | --- | --- | --- |
| ABM with ongoing MDD | 27.5 | 20.6 | 21.3 |
| ABM without ongoing MDD | 18.7 | 13.3 | 16.6 |
| Sham training with ongoing MDD | 30.0 | 24.5 | 21.0 |
| Sham training without ongoing MDD | 23.8 | 15.4 | 13.6 |

*Note.* ABM = Attention bias modification, BDI-II = Becks Depression Inventory-II, MDD = Major depressive disorder.

### Comorbid anxiety

To investigate if comorbid anxiety was relevant to the treatment effect, we added comorbid anxiety as predictor to the analysis of the primary outcome. The interaction effects of the primary outcome analysis remained unchanged, however, there was now a main effect of ABM condition, t (143.0747) = -2.11, *p* = .04, *d =* .35. There was no interaction effect between ABM and comorbid anxiety, t (143.6217) = 1.66, *p* = .10, *d =* .28. There was not a significant three-way interaction effect between ABM, comorbid anxiety, and assessment point from baseline to post-treatment t (147.9513) = -.77, *p* = .44, *d* = .13, nor from baseline to six months follow up t (148.1526) = -.09, p = .37, *d* = .015, or from post-treatment to six months follow-up, t (146.56328) = -.134, *p* = .9, *d* = .02. There was no significant difference between groups at six-months follow-up, F (3,75) = 1.05, *p* = .38, *η^2^* = .04. Hence, presence of comorbid anxiety disorders did not moderate the association between ABM and depressive symptoms at any of the assessment points, however, including comorbid anxiety as predictor revealed a significant main effect of ABM, where depression levels were lower among the ABM group compared to the group receiving the sham condition.

***Supplementary table 2.***

Estimated marginal means of depression severity as measured by BDI-II separated by ongoing comorbid anxiety disorder.

|  | Baseline | Post-intervention | Six-months follow-up |
| --- | --- | --- | --- |
| ABM with comorbid anxiety | 26.4 | 18.2 | 17.6 |
| ABM without comorbid anxiety | 18.2 | 14.7 | 19.8 |
| Sham training with comorbid anxiety | 26.0 | 19.3 | 14.9 |
| Sham training without comorbid anxiety | 27.0 | 20.5 | 20.0 |

*Note.* ABM = Attention bias modification, BDI-II = Becks Depression Inventory-II.

***Supplementary table 3.***

Estimated marginal means of anxiety severity as measured by BAI.

|  | Baseline | Post-intervention | Six-months follow-up |
| --- | --- | --- | --- |
| ABM | 12.5 | 9.9 | 12.0 |
| Sham training | 16.2 | 12.5 | 10.4 |

*Note.* ABM = Attention bias modification, BAI = Beck’s anxiety inventory.
